# Charge Transport in Organic Semiconductors: The Perspective from Nonadiabatic Molecular Dynamics

**DOI:** 10.1021/acs.accounts.1c00675

**Published:** 2022-02-23

**Authors:** Samuele Giannini, Jochen Blumberger

**Affiliations:** † Department of Physics and Astronomy and Thomas Young Centre, University College London, London WC1E 6BT, United Kingdom

## Abstract

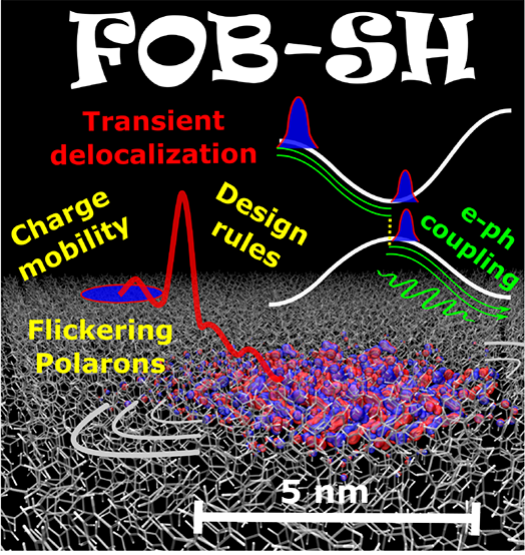

Organic semiconductors (OSs) are an exciting class of materials that have enabled disruptive technologies in this century including large-area electronics, flexible displays, and inexpensive solar cells. All of these technologies rely on the motion of electrical charges within the material and the diffusivity of these charges critically determines their performance. In this respect, it is remarkable that the nature of the charge transport in these materials has puzzled the community for so many years, even for apparently simple systems such as molecular single crystals: some experiments would better fit an interpretation in terms of a localized particle picture, akin to molecular or biological electron transfer, while others are in better agreement with a wave-like interpretation, more akin to band transport in metals.

Exciting recent progress in the theory and simulation of charge carrier transport in OSs has now led to a unified understanding of these disparate findings, and this Account will review one of these tools developed in our laboratory in some detail: direct charge carrier propagation by quantum-classical nonadiabatic molecular dynamics. One finds that even in defect-free crystals the charge carrier can either localize on a single molecule or substantially delocalize over a large number of molecules depending on the relative strength of electronic couplings between the molecules, reorganization, or charge trapping energy of the molecule and thermal fluctuations of electronic couplings and site energies, also known as electron–phonon couplings.

Our simulations predict that in molecular OSs exhibiting some of the highest measured charge mobilities to date, the charge carrier forms “flickering” polarons, objects that are delocalized over 10–20 molecules on average and that constantly change their shape and extension under the influence of thermal disorder. The flickering polarons propagate through the OS by short (≈10 fs long) bursts of the wave function that lead to an expansion of the polaron to about twice its size, resulting in spatial displacement, carrier diffusion, charge mobility, and electrical conductivity. Arguably best termed “transient delocalization”, this mechanistic scenario is very similar to the one assumed in transient localization theory and supports its assertions. We also review recent applications of our methodology to charge transport in disordered and nanocrystalline samples, which allows us to understand the influence of defects and grain boundaries on the charge propagation.

Unfortunately, the energetically favorable packing structures of typical OSs, whether molecular or polymeric, places fundamental constraints on charge mobilities/electronic conductivity compared to inorganic semiconductors, which limits their range of applications. In this Account, we review the design rules that could pave the way for new very high-mobility OS materials and we argue that 2D covalent organic frameworks are one of the most promising candidates to satisfy them.

We conclude that our nonadiabatic dynamics method is a powerful approach for predicting charge carrier transport in crystalline and disordered materials. We close with a brief outlook on extensions of the method to exciton transport, dissociation, and recombination. This will bring us a step closer to an understanding of the birth, survival, and annihiliation of charges at interfaces of optoelectronic devices.

## Key References

- GianniniS.; CarofA.; EllisM.; YangH.; ZiogosO. G.; GhoshS.; BlumbergerJ.Quantum localization and delocalization of charge carriers in organic semiconducting crystals. Nat. Commun.2019, 10, 38433145168710.1038/s41467-019-11775-9PMC6710274.^1^*Nonadiabatic molecular dynamics simulations on a set of organic crystals show that thermal intraband excitations from modestly delocalized band edge states to highly delocalized tail states give rise to quantum delocalization of the charge carrier wave function that drives polaron diffusion.*
- GianniniS.; CarofA.; BlumbergerJ.Crossover from hopping to band-like charge transport in an organic semiconductor model: Atomistic non-adiabatic molecular dynamics simulation. J. Phys. Chem. Lett.2018, 9, 3116–31232978727510.1021/acs.jpclett.8b01112PMC6077769.^2^*Nonadiabatic molecular dynamics simulations are carried out for a 1D molecular model system to probe the crossover from hopping to band-like transport as a function of temperature and electronic coupling.*
- GianniniS.; ZiogosO. G.; CarofA.; EllisM.; BlumbergerJ.Flickering Polarons Extending over Ten Nanometres Mediate Charge Transport in High-Mobility Organic Crystals. Adv. Theory Simul.2020, 3, 2000093.^3^*The mobility tensor is calculated for 2D planes of molecular organic crystals and compared to experimental data. The transient delocalization scenario of the charge carrier is described in detail.*
- EllisM.; YangH.; GianniniS.; ZiogosO. G.; BlumbergerJ.Impact of Nanoscale Morphology on Charge Carrier Delocalization and Mobility in an Organic Semiconductor. Adv. Mater.2021, 33, 210485210.1002/adma.202104852PMC1146904934558126.^4^*Quantum dynamical simulations of hole transport in disordered phases of pentacene show a clear correlation between the crystallinity of the sample, the quantum delocalization and the mobility of the charge carrier.*

## 1 Introduction

Organic semiconductors (OSs) combine many advantages that make them attractive for a host of different applications, e.g., easy chemical tunability, synthesis from renewable materials free of nonprecious elements, mechanical flexibility, and light weight. However, this comes at the cost of relatively limited charge mobilities compared to inorganic semiconductors: to date, the highest reproducible mobilities in molecular and polymeric OSs is ca. 20 cm^2^ V^–1^ s^–1^ for holes and ca. 8–9 cm^2^ V^–1^ s^–1^ for electrons,^5^ which exceeds the one for amorphous silicon (≈ 1 cm^2^ V^–1^ s^–1^) but falls short of the mobility for single crystalline silicon (≈ 10^3^ cm^2^ V^–1^ s^–1^). Developing easily processable organic materials with higher charge mobility values is highly desirable as it would, e.g., allow for higher switching frequencies between on and off states in organic transistors, lead to smaller resistance and decreased power consumption, and improve the efficiency of organic solar cells by reducing charge recombination.

The search and identification of OSs with (ultra)high charge mobilities is likely to benefit from a better fundamental understanding of charge carrier transport in OSs. Experimentally, one often places reliance on the temperature dependence of mobility to infer how charges might move through the material.^5−7^ A decrease of charge mobility (μ) with increasing temperature (*T*) has often been reported for high-quality single-crystal devices from time-of-flight experiments,^8^ time-resolved terahertz pulse spectroscopy,^9^ and space-charge-limited current measurements.^10^ The temperature dependence could often be described by a power law, μ ∝ *T*^–*n*^ with 0.5 < *n* < 3, and it was interpreted in terms of a band-like transport^11,12^ in accord with the situation in metals. On the other hand, results from charge modulation spectroscopy^13,14^ were in better agreement with charge carriers being localized on a few molecules implying that a charge hopping mechanism may be a suitable description. Other experiments have also been interpreted in terms of a coexistence of localized and more delocalized states in OSs.^15,16^

**Figure 1:**
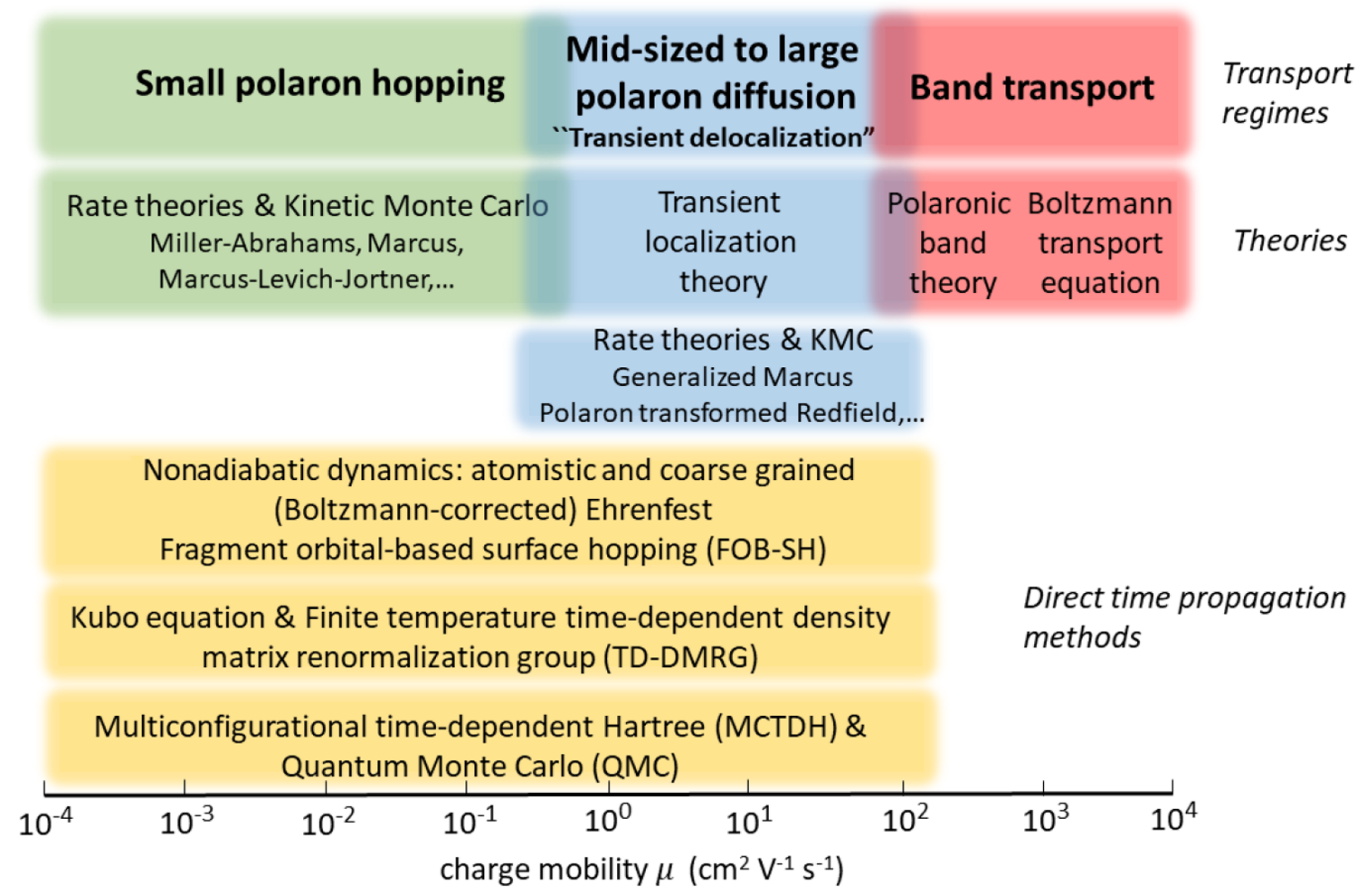
Charge transport regimes for organic semiconductors. Analytic charge transport theories and their range of validity (green, blue, red) and direct time propagation methods.

From a theoretical point of view, the band and charge hopping regimes are extreme cases which almost never apply in (defect free) molecular OSs;^11,17,18^ see Figure 1. Most OSs exhibiting a decent charge mobility (>0.1 cm^2^ V^–1^ s^–1^) are in a difficult transport regime that is “between” these extreme limits and that has eluded a theoretical description for some time, though elegant polaronic theories were proposed to account for localization of the carrier and band narrowing with increasing temperature as a consequence of the larger polaron mass and electron–phonon scattering.^12,19,20^ However, like band theory, polaronic band theories also become problematic at higher temperatures.^11^ The last 10 years have witnessed exciting progress in the development of theoretical and computational approaches that have shed new light on this important charge transport regime which is now widely referred to as “Transient Localization Scenario”.^17^ However, for reasons that will become clear in this Account, we prefer to call it “Transient Delocalization Regime”.^3^ These methods include, e.g., transient localization theory (TLT),^7,17,21,22^ delocalized charge carrier hopping based on generalized Marcus theory^23^ or polaron-transformed Redfield theory^24^ mapped onto kinetic Monte Carlo,^25^ Kubo formula solved by finite temperature time-dependent density matrix renormalization group (TD-DMRG),^26,27^ or the mobility relation with the imaginary-time current–current correlation function solved using quantum Monte Carlo techniques (QMC).^28^ Finally, direct charge propagation based on nonadiabatic dynamics schemes using either a fully atomistic^2,29−35^ or a coarse grained description of the nuclear degrees of freedom^36−39^ has been used to predict mobility and wave function delocalization in OSs; see Figure 1 for a summary.

In this Account, we place focus on our own contribution, the development^2,33−35,40,41^ and application^1,3,4,42^ of a direct charge propagation scheme in the framework of nonadiabatic molecular dynamics, termed fragment orbital-based surface hopping, short FOB-SH. In the next section, we briefly describe FOB-SH and then present applications of the method to charge transport in a 1D model system, in single crystalline and disordered molecular OSs. We summarize and discuss the design rules that should pave the way toward new ultrahigh mobility OSs and explain why covalent organic frameworks are a promising class of materials in this respect.

## 2 Theory and Methodology

### 2.1 Fragment Orbital-Based Surface Hopping (FOB-SH)

**Figure 2:**
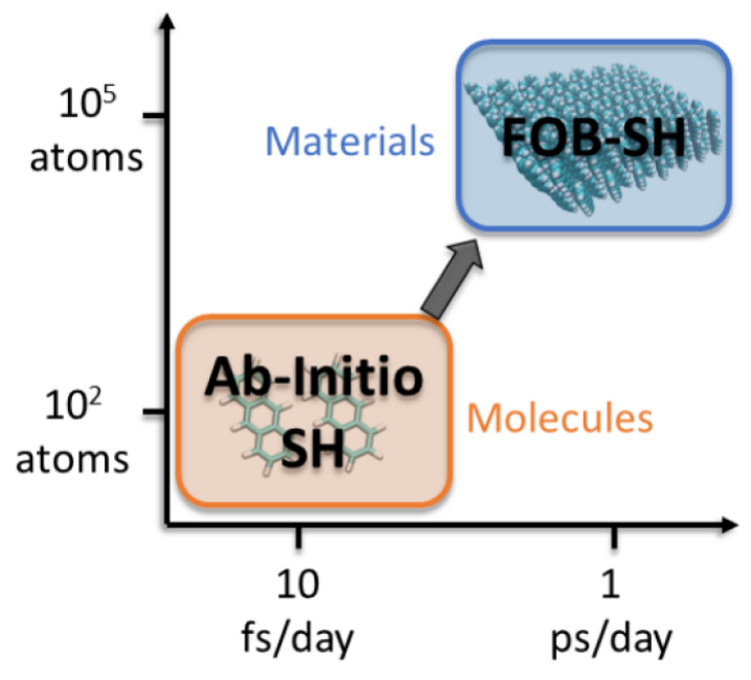
From molecules to materials. Time scales and system sizes currently accessible with ab initio or TDDFT-based fewest switches surface hopping (“Ab-initio SH”) and with fragment orbital-based surface hopping (“FOB-SH”). Note, time scales and system sizes are just indicative, as they depend on the level of theory used.

FOB-SH solves the time-dependent electronic Schrödinger equation for an electron hole in the valence band (or for an excess electron in the conduction band) of a molecular material subject to the thermal motion of the nuclei.^2,33−35,41^ The latter is treated in accord with Tully’s fewest switches surface hopping algorithm.^43^ FOB-SH is computationally very efficient pushing nonadiabatic dynamics from the molecular to the true nanoscale (>10 nm); see Figure 2. In the following, we briefly sketch the basic theory and refer to a recent book chapter^41^ and other technical papers^2,33−35^ for a detailed description of the FOB-SH method.

**Figure 3:**
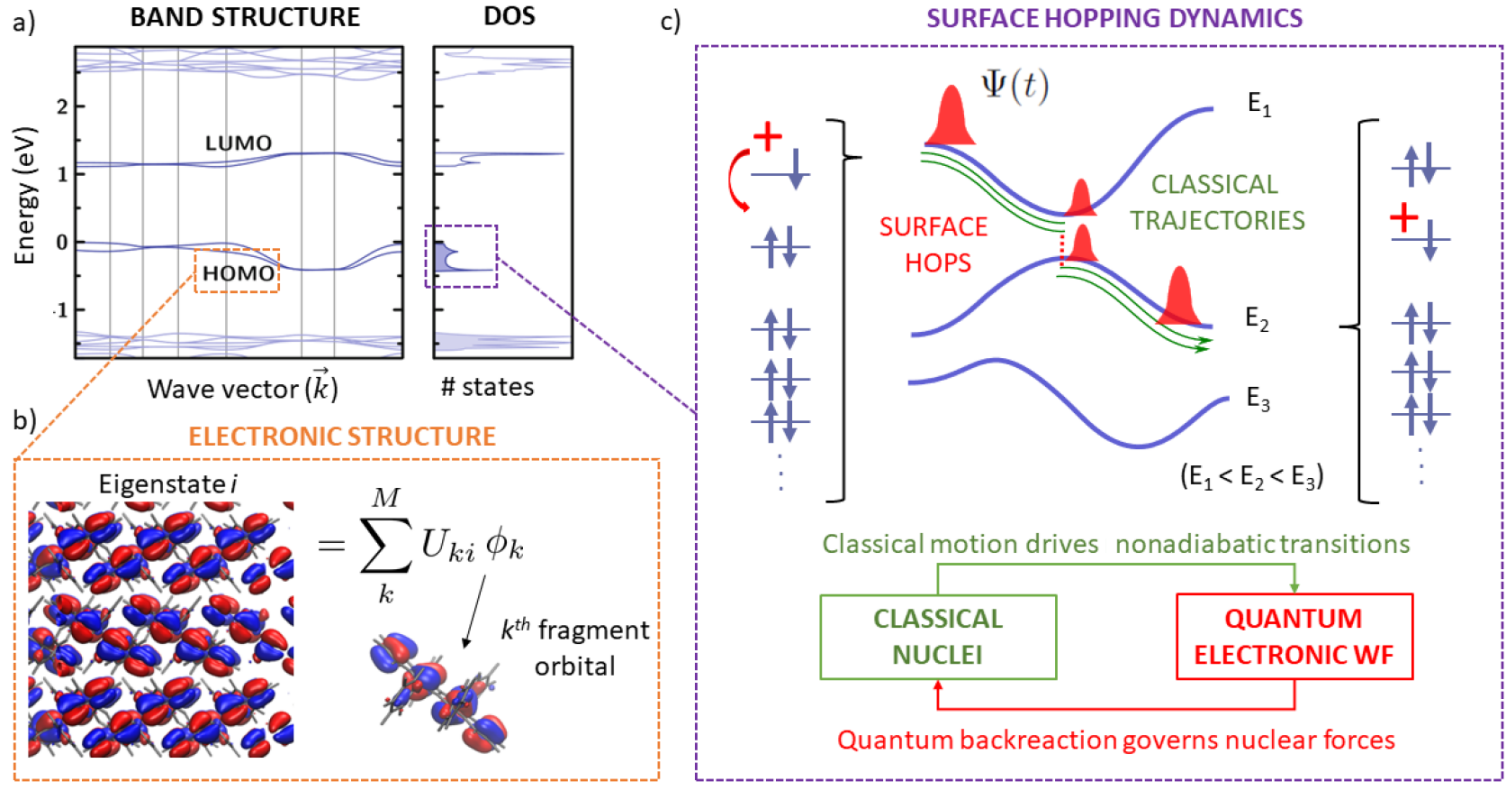
Fragment orbital-based surface hopping (FOB-SH) in a nutshell; see section 2.1 for an explanation.

The interaction between the molecules forming a solid is relatively weak which gives rise to a modest band dispersion (≈ 0.1–0.5 eV) as compared to conventional inorganic semiconductors; see Figure 3a. Hence, the highest valence (lowest conduction) band states of these materials can be approximated by a linear combination of the highest occupied molecular orbital or HOMO (lowest unoccupied molecular orbitals or LUMO) of the constituent molecules;^11,44^Figure 3b. FOB-SH takes advantage of this by approximating the complicated many-body electronic dynamics by a one-particle wave function for the excess charge, Ψ(*t*), written as a linear combination of the orthogonalized HOMOs (LUMOs), which we call more generally fragment orbitals {ϕ_*l*_(**R**(*t*))}:
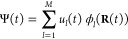
1where *u*_*l*_ are the expansion coefficients of each fragment orbital contributing to Ψ(*t*). Insertion of eq 1 in the time-dependent Schrödinger equation gives the time-evolution of the charge carrier wave function in the valence (conduction) band of the semiconductor,
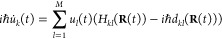
2where *d*_*kl*_ = ⟨ϕ_*k*_|ϕ̇_*l*_⟩ are the nonadiabatic coupling elements and *H*_*kl*_ = ⟨ϕ_*k*_|*H*|ϕ_*l*_⟩ is the electronic Hamiltonian of the system in the basis of fragment orbitals. *H*_*kk*_ is also called site energies, and *H*_*kl*_, *k* ≠ *l*, electronic couplings between molecules *k* and *l*.^33−35,41^

The nuclear degrees of freedom, **R**(*t*), are propagated on one of the potential energy surfaces (PES) obtained by diagonalizing the electronic Hamiltonian (see Figure 3c), denoted as *E*_a_ (“a” for “active surface”). Nuclear forces are obtained using the Hellmann–Feynman theorem; see refs (33) and (34) for details. The nuclear motion on the PES couples to the motion of the excess charge carrier via the dependences of all electronic Hamiltonian matrix elements *H*_*kl*_ in eq 2 on the nuclear positions (**R**(*t*)). This results in thermal fluctuations of the site energies and electronic couplings, also denoted local and nonlocal electron–phonon coupling, respectively. Feedback from the motion of the excess charge carrier to the nuclear dynamics is accounted for by nonadiabatic transitions (“hops”) from the PES of the active eigenstate *a* to the PES of another eigenstate *j* using Tully’s surface hopping probability;^43^ see Figure 3c. A swarm of independent classical trajectories is then propagated in time to represent the dynamics of a quantum wave packet (i.e., wave packet superposition and branching effects).

To open up applications to truly nanoscale materials, we employ an electronic Hamiltonian that is parametrized against DFT calculations and updated on-the-fly during the time propagation without performing explicit electronic structure calculations, thus surpassing ab initio or TDDFT-based surface hopping schemes^45,46^ in terms of accessible system sizes and time scales; see Figure 2. In particular, the site energies *H*_*kk*_ and gradients ∇_**R**_*H*_*kk*_ are approximated with parametrized classical force fields, while electronic couplings *H*_*kl*_, coupling derivatives ∇_**R**_*H*_*kl*_, and *d*_*kl*_ between the fragment orbitals are computed along the dynamics using a very efficient analytic overlap method (AOM).^47^ An accurate evaluation of the transport parameters (i.e., reorganization energies, electronic couplings, and their fluctuations) is very important for the calculation of transport properties. In our method, classical force fields are parametrized to reproduce DFT reorganization energies obtained using the B3LYP hybrid functional, which in turn was shown to accurately reproduce experimental reorganization energies from UPS measurements.^48,49^ AOM electronic couplings were parametrized against DFT electronic couplings which in turn were validated against high-level ab initio methods.^50−52^ For an accurate dynamics, the surface hopping algorithm also needs to be supplemented with a number of important features: decoherence correction, trivial crossing detection, elimination of spurious long-range charge transfer, and adjustment of the velocities in the direction of the nonadiabatic coupling vector in the case of a successful surface hop. We refer to refs (34), (35), and (41) for a detailed description and discussion of the importance of these additions to the original fewest switches surface hopping method.^43^

It is worth noting that FOB-SH is a nonperturbative method that goes beyond the linear electron–phonon coupling approximation, which is often assumed in model Hamiltonians (Holstein or Holstein–Peierl), and it incorporates nonadiabatic nuclear dynamics effects, which are usually missing in analytic charge transport theories. Moreover, it encompasses a wide range of mechanistic regimes, from small polaron hopping to large polaron diffusion, see Figure 1.

FOB-SH also has limitations. The dynamics of the individual trajectories is classical; i.e., the nuclei follow the classical equations of motion. Thus, nuclear-quantum effects such as nuclear tunneling and zero point energy are missing. In a first attempt to overcome this drawback, our group^40^ has combined FOB-SH with path integrals, specifically ring-polymer molecular dynamics.^53,54^ Applications to a model donor–acceptor system with parameters representative for OSs showed that nuclear quantum effects lead to an increase in the transfer rate by more than an order of magnitude at low temperature while the effect turned out to be very modest at room temperature. More generally, we expect that nuclear tunneling and zero-point motion are important for low temperature in low mobility materials with significant energy barriers (small polaron hopping regime), but relatively unimportant at room temperature in high mobility materials where no barriers are present (large polaron diffusion regime). While our fully atomistic scheme can be straightforwardly extended to treat other electronic phenomena, e.g., exciton transport, exciton splitting and charge recombination, in this work we place focus solely on charge carrier transport in a monocomponent crystalline and/or amorphous phase. Moreover, carrier–carrier interactions are not included, i.e. transport at low charge carrier concentration is modeled.

### 2.2 Charge Mobility and IPR

The time-dependent charge carrier wave function, Ψ(*t*) in eq 1, gives access to key dynamical properties, e.g., the mobility tensor (eq 3), the extent of delocalization of the charge carrier, and the mechanism by which the charge carrier moves within the material. The charge mobility may be expressed as a second rank tensor using the Einstein relation
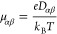
3where α(β) represents *x*, *y*, *z* Cartesian coordinates, *e* is the elementary charge, *k*_B_ is the Boltzmann constant, and *T* is the temperature. The diffusion tensor components, *D*_αβ_, can be obtained as the time derivative of the mean squared displacement along the nine Cartesian components (MSD_αβ_)^35,41^ from FOB-SH simulations: .

The MSD_αβ_ is calculated as follows:
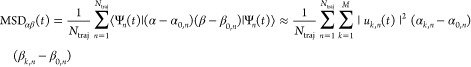
4where Ψ_*n*_(*t*) is the time-dependent charge carrier wave function in trajectory *n*, α_0,*n*_(β_0,*n*_) are the initial positions of the center of charge in trajectory *n*, α_0,*n*_ = ⟨Ψ_*n*_(0)|α|Ψ_*n*_(0)⟩, and the square displacements are averaged over *N*_traj_ FOB-SH trajectories. In the second equation, the coordinates of the charge are discretized and replaced by the center of mass of molecule *k* in trajectory *n*, α_*k*,*n*_, and α_0,*n*_ = ∑_*k*=1_^*M*^|*u*_*k,n*_(0)|^2^α_*k,n*_(0), where |*u*_*k,n*_(*t*)|^2^ is the time dependent charge population of site *k* in trajectory *n* as obtained by solving eq 2.

A common measure for the delocalization of the charge carrier wave function Ψ(*t*) is the inverse participation ratio (IPR).^35,41,55^ The numerical value of the IPR is a measure for the number of molecules over which the wave function is delocalized.

## 3 Applications

### 3.1 Crossover from Hopping to Band-like Transport in a 1D Model

In a first application of the method we investigated whether FOB-SH can predict the crossover from hopping to band-like transport, which has been a widely visited and debated topic in the literature.^5−7,17^ The advantage of nonperturbative schemes like FOB-SH is that the transport mechanism can be readily obtained from the simulation, without reference to the temperature dependence.

**Figure 4:**
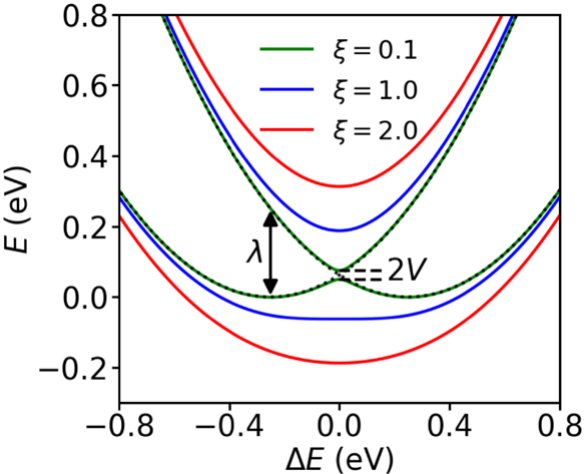
Potential energy curves for electron transfer in a two-state donor–acceptor system. ξ = 2*V*/λ, with *V* being the mean electronic coupling and λ being the reorganization energy. See section 3.1 for details.

We investigated a 1D atomistic model of an OS, a chain of ethylene-like molecules (ELMs).^2^ The term “ethylene-like” stresses that only the nuclear geometries correspond to a real ethylene molecule, while the charge transport parameters, i.e. reorganization energy (λ) and mean electronic coupling between neighboring pairs (*V* = ⟨|*H*_*kl*_|^2^⟩^1/2^), are chosen freely to explore different transport regimes. To this end, we define the reduced coupling strength between the molecules, ξ = 2*V*/λ. For a simple two-state (donor–acceptor) system, if ξ < 1, a finite energy barrier (maximum) between the two charge localized states (minima) exists (green curve in Figure 4). If ξ = 1, the barrier vanishes and the two charge localized states are no longer stable (blue curve in Figure 4). For ξ > 1, the barrier becomes a minimum (red curve in Figure 4). An analog to this is the Robin–Day classification of mixed valence compounds.

**Figure 5:**
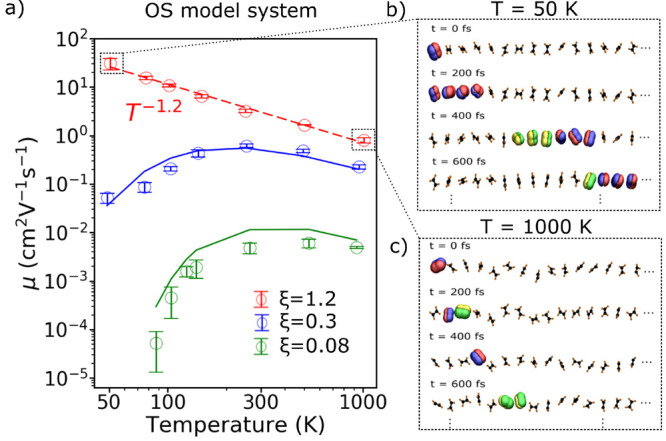
Crossover from hopping to band-like transport in a 1D model of an OS, as obtained from FOB-SH simulation (empty circles). Mobilities obtained by solving chemical Master equation for nearest neighbor hopping are reported with solid green and blue lines. See section 3.1 for details. Reproduced from ref (2). Copyright 2018 American Chemical Society.

In Figure 5a, we report the FOB-SH hole mobility along a chain of ethylene-like molecules for ξ = 0.08, 0.3, 1.2 denoted in the following as “small”, “medium”, and “large” coupling strength regime.^2^ For small and medium coupling strengths, we observe thermally activated transport at low temperatures up to room temperature. For larger temperatures, thermal energy becomes comparable or larger than activation energy, the hopping rates and diffusion constants saturate with temperature (i.e., *D* becomes *T*-independent at high temperatures), and the mobility decreases due to the *T*^–1^ dependence of the mobility; see eq 3. As the coupling strength is increased, the activated regime at low temperature gradually crosses over to a band-like decay. For the largest coupling strength, the mobility exhibits a band-like decrease for all temperatures according to a power law μ ∝ *T*^–1.2^. Similar trends with temperature have been reported in experiments^5−10^ as well as in previous simulations, where a coarse grained model Hamiltonian was employed.^55,56^

The transport mechanism in the different regimes can be readily investigated by analyzing the time-dependent wave function Ψ(*t*). We find that for small coupling strength (ξ = 0.08, finite barrier) the hole is localized on a single molecule and moves along the chain by nearest-neighbor hops. Indeed, hole mobilities obtained from Marcus rates are found to be in very good agreement with FOB-SH mobilities in this regime (solid green and blue curves in Figure 5a). Remarkably, the hopping transport occurs at all temperatures, even in the apparent “band-like” regime at high temperatures. Thus, “band-like” has nothing to do with band transport here but merely arises from the *T*^–1^ dependence of the mobility, eq 3, as previously mentioned.

The situation is strikingly different at high coupling strength (ξ = 1.2, no barrier), as illustrated in Figure 5b and c where we show Ψ(*t*) along a randomly selected FOB-SH trajectory. At low temperatures, the hole is delocalized over about four ELMs and quickly moves along the chain (panel b). As the temperature increases, the delocalization of the polaron decreases to 2 and the transport along the chain becomes more sluggish (panel c). Again, what we call “band-like” has nothing to do with band transport but points to the fact that polaron size, and with it the mobility, decreases with increasing temperature. Evidence from many experimental measurements has also pointed to the existence of finite size polarons typically localized on a few molecular sites.^13,14,57,58^

### 3.2 Charge Transport in Organic Single Crystals

#### 3.2.1 Computed vs Experimental Mobilities

**Figure 6:**
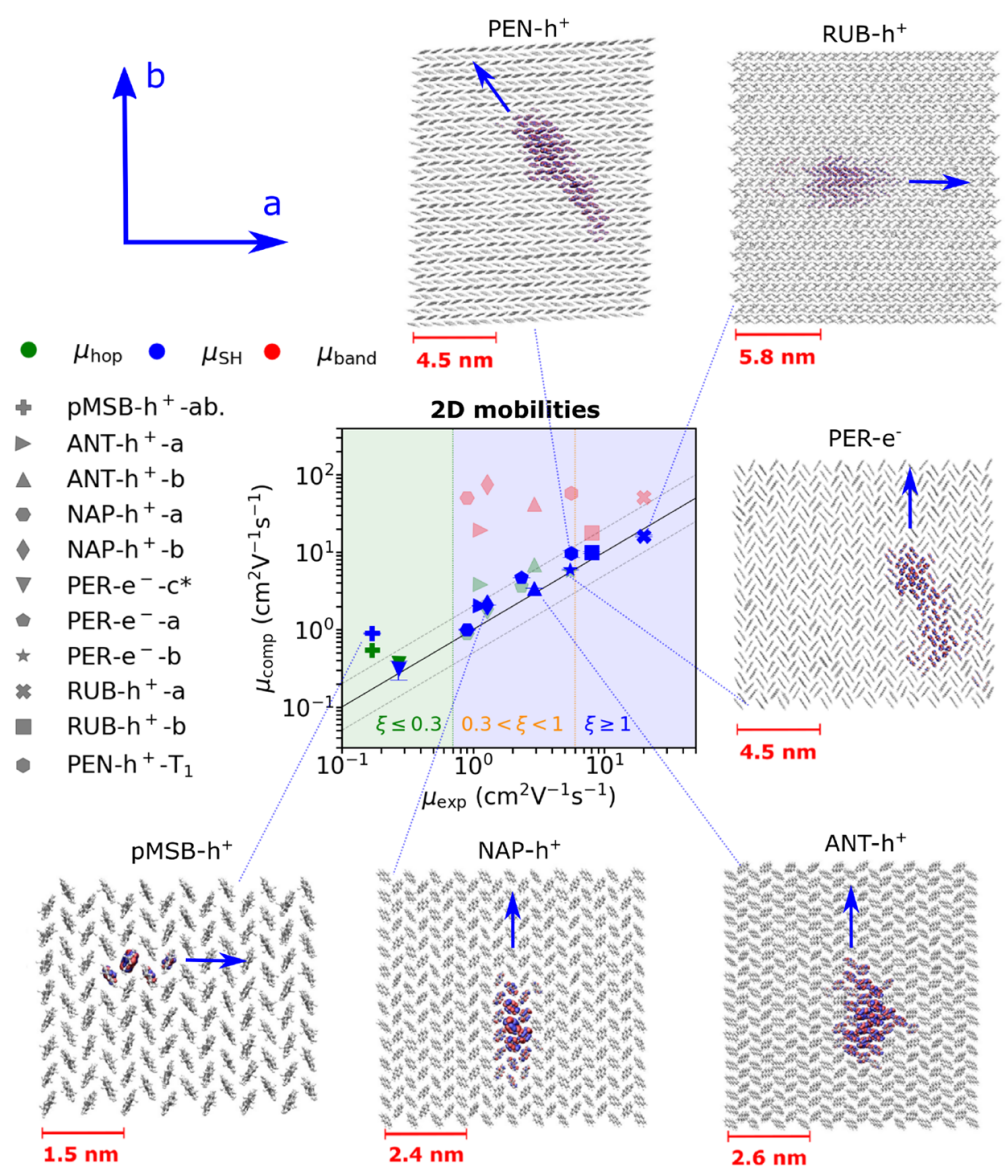
Computed versus experimental charge mobilities and polaron delocalization for a set of molecular organic single crystals. Charge mobilities from FOB-SH are given in blue, mobilities obtained by solving a chemical Master equation for nearest neighbor hopping are in green,^3^ and band theory data (taken from the literature) are depicted in red. ANT-h^+^-a denotes hole transport along crystallographic direction *a* for anthracene, and similarly for the other crystals. See section 3.2 for further details. Adapted from ref (3) with permission from Wiley.

The FOB-SH method is sufficiently efficient to be used for the calculation of charge mobilities in realistic nanoscale samples of OSs (see Figure 2). We have benchmarked the accuracy of the method by computing the room temperature hole or electron mobility in the conductive layers (*a*–*b* planes) of experimentally well-characterized molecular organic crystals such as 1,4-bis(4-methylstyryl)benzene (pMSB), naphthalene (NAP), anthracene (ANT), perylene (PER), pentacene (PEN), and rubrene (RUB), as reported in Figure 6 (data in blue) and summarized in Table 1. We find that after a short initial relaxation period the mean squared displacement of the charge carrier increases approximately linearly with time for all systems, indicative of Einstein diffusion.^3^ The charge mobilities are obtained using eq 3. For orthorhombic and monoclinic crystals the Cartesian coordinates (*x*, *y*) of the supercell were chosen parallel to the crystallographic directions (*a*, *b*) that define the high mobility plane. In this representation, the off-diagonal components of the diffusion tensor are zero due to symmetry, and one can consider just the diagonal tensor components (along *a* and *b*). For pentacene (triclinic) the diffusion tensor was diagonalized. The convergence of mobility with respect to system size and time step was investigated thoroughly in ref (3). We found that a time step of about 0.01 fs is required for low mobility OSs to deal effectively with the so-called trivial crossings problem affecting localized charges and a larger time step of 0.05 fs can be used for high mobility OSs. System sizes of one to a few hundred molecules are sufficient for low mobility OSs, whereas sizes of up to 1000 molecules are required for high mobility OSs to remove as much as possible the boundary effects of the finite simulation cell on diffusion. The mobilities span 3 orders of magnitude and most fall well within the uncertainties of experimentally determined values. By contrast, band theory systematically overestimates mobilities (red symbols),^59−61^ and while the small polaron hopping model (green symbols) gives a good description for low mobilities, they become inapplicable for high-mobility systems like pentacene and rubrene, where ξ > 1.

**Table 1:** Comparison between FOB-SH Mobilities and IPR Obtained from Simulation of the Full 2D Planes (Ref (3) μ (2D)) and from Reduced 1D Models along the Specified Directions within the 2D Planes (Ref (1) μ (1D)), and Experimental Results (μ (exp))^a^

	dir.	μ (1D)	μ (2D)	μ (exp)	IPR (1D)	IPR (2D)
pMSB	*a*		1.1	0.17^b^^,^^c^		1.7
	*b*	0.21	0.61		1.1	
NAP	*a*		1.0	0.9^c^		2.5
	*b*	1.3	2.1	1.3^c^	1.6	
ANT	*a*	0.86	2.0	1.1^c^	2.0	5.0
	*b*	1.9	3.5	2.9^c^	2.2	
PER	*a*	2.4	4.7	2.3^c^^,^^d^	1.7	12
	*b*		6.0	5.5^c^^,^^d^		
RUB	*a*	4.9	16	9.6,^c^ 15,^c^ 20^c^	3.2	14
	*b*		10	3.7,^c^ 4.4,^c^ 7.8^c^		
PEN	*T*_1_^⊥′^		0.92^e^	5.0,^b^^,^^c^ 5.6,^b^^,^^c^ 11^b^^,^^c^		17^f^
	*T*_1_^′^	9.6	9.6^e^		6.8	

a All values for mobility are in cm^2^ V^–1^ s^–1^.

b Transport direction unknown.

c Experimental reference is given in the Supporting Information of ref (3).

d For this system electron mobility was simulated.

e Mobilities along the eigendirections of the 2D mobility tensor, *T*_1_^′^ and *T*_1_^⊥′^.

f In ref (57) the wave function delocalization was estimated to be 17 molecules using ESR spectroscopy.

#### 3.2.2 Polaron Delocalization and Transport Regimes

The insets in Figure 6 show a representative snapshot of the charge carrier wave function Ψ(*t*) for each system. It is immediately apparent that charge mobility correlates well with the extent of charge carrier delocalization, the central result of TLT.^7,17,22^ Moreover, the anisotropy in the spatial extension of the charge carrier correlates with the anisotropy in mobility and electronic couplings. In the OS that exhibits the smallest mobility (pMSB, μ < 1 cm^2^ V^–1^ s^–1^), the barrier is significant (ξ ≈ 0.1), causing the charge carrier to localize on a single molecule. Charge propagation occurs via thermally activated small polaron hopping events. For OSs with larger mobilities (naphtalene, anthracene, perylene, μ ≈ 1–5 cm^2^ V^–1^ s^–1^), the barrier is small (0.3 < ξ < 1), causing the polaron to delocalize over a modest number of molecules, about 3–10. We call this regime midsized polaron diffusion. Evidently, the small polaron hopping model is no longer physically justified in this regime and the agreement of the hopping mobility with experiments for these systems must be considered a coincidence. For OSs that exhibit even higher mobilities (pentacene, rubrene, μ ≥ 5 cm^2^ V^–1^ s^–1^), the energy barrier has vanished (ξ ≥ 1) and polarons are delocalized over a substantial number of molecules. For pentacene, we obtain on average 17–18 molecules, in excellent agreement with an estimate of 17 from experimental electron spin paramagnetic resonance data at 290 K.^57^ As the polaron size, about 5 nm radius, reaching up to 10 nm during a transient delocalization event (see below), is significantly larger than the unit cell dimensions, we call this regime large polaron diffusion. Notice that in this regime the finite size of the polaron is due to the thermal fluctuations (disorder) of electronic couplings, also termed nonlocal electron–phonon coupling, and fluctuations of the site energies, also called local electron–phonon coupling.

#### 3.2.3 Zooming into the Transient Delocalization Mechanism

**Figure 7:**
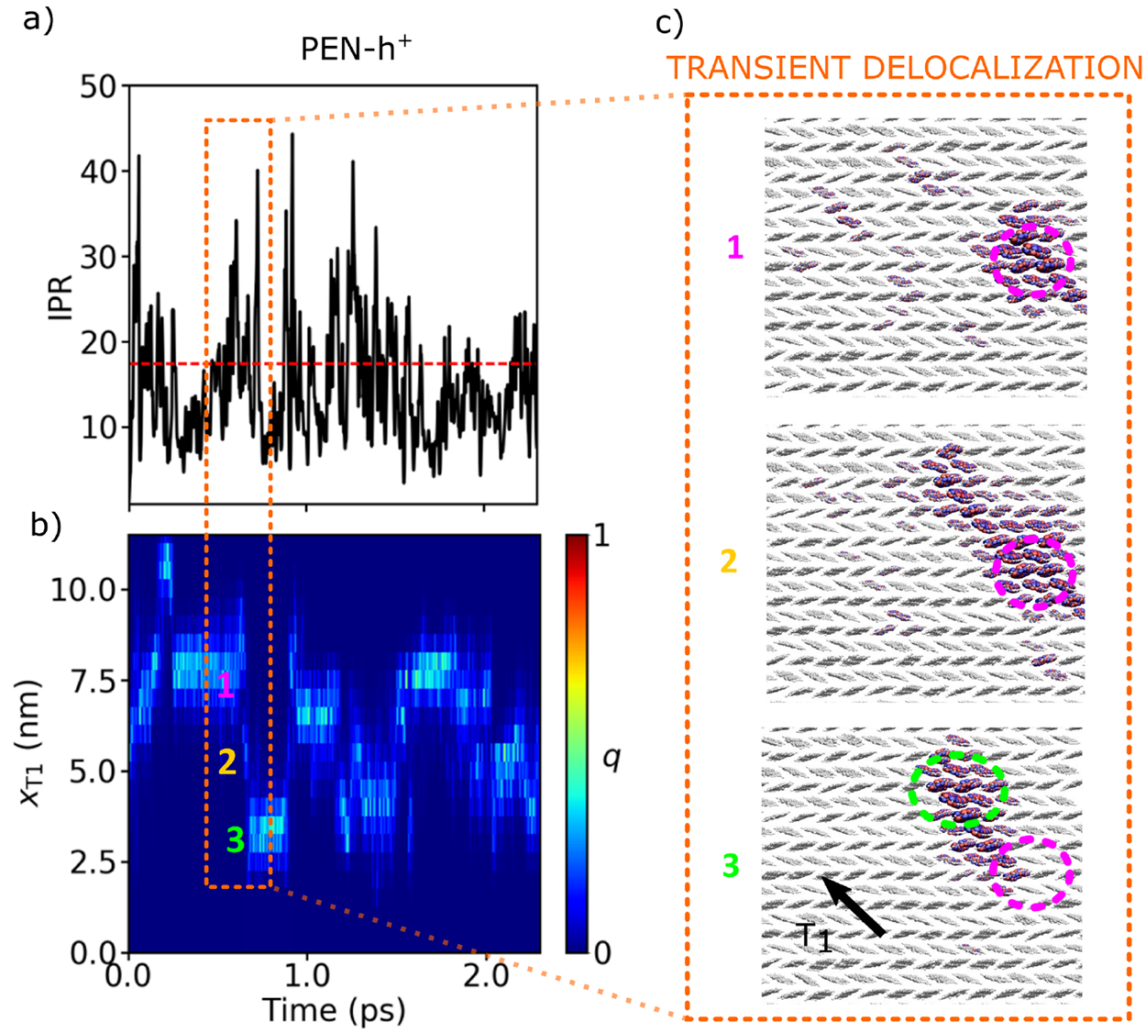
Time evolution of the hole wave function in a pentance single crystal, along a representative FOB-SH trajectory. (a) Inverse participation ration (IPR) with mean value indicated in dashed red lines. (b) Molecular amplitudes of the hole wave function (|*u*_*k*_|^2^, eq 1) projected onto the *T*_1_ crystallographic direction, *x*_T1_. The integral amplitudes (*q*) increase from dark blue to light blue. (c) Transient delocalization event resulting in the spatial displacement of the hole wave function (repositioned here for illustrative purpose). See section 3.2 for further details. Adapted from ref (1) with permission from Nature Publishing Group.

The transport scenario in the midsized and large polaron diffusion regimes have widely been termed “transient localization”.^7,17,22^ However, if we follow a typical time evolution of the charge carrier wave function, Ψ(*t*), we find it more appropriate to call the mechanism transient delocalization, see, e.g., Figure 7 for pentacene. Ψ(*t*) populates most of the time energetically low-lying hole states close to the top of the valence band edge, which are delocalized over 10–20 molecules (about 5–6 nm); see IPR in Figure 7 panel (a), horizontal sections in panel (b), and the wave function snapshot in the top panel (c). The polaron is a highly dynamic object; its size and spatial extensions strongly fluctuate in time (see panel (a)), which is why we dubbed it “flickering polaron”.^3^ In some instances, short-lived thermal intraband excitations to more delocalized states closer to the middle of the valence band occur leading to a significant expansion of the carrier wave function to about twice its average size (IPR = 30–40; see spikes in Figure 7 panel (a) and the snapshot in the middle panel in (c)). Some of these sudden expansions of the wave function are successful, meaning Ψ(*t*) localizes over the cluster of molecules to which it expanded and at the same time this cluster becomes an energetically low-lying hole eigenstate close to the top of the valence band (see Figure 7 panel (b) and lower panel (c)). These expansion–contraction events, which we also termed “diffusive jumps” in resemblance with molecular diffusion in heterogeneous media,^62^ spatially displace the polaron by a distance that is on the order of its diameter and give rise to diffusion, charge mobility, and electronic conduction. The nature of the polaron expansions is transient, on the order of 10–15 fs (see peaks in panel (a)), while the duration between these events is about 100–500 fs depending on the material, the typical time scale of electronic coupling fluctuations (corresponding to the relaxation time τ in transient localization theory). In reference to these time scales, we refer to this mechanism as “transient delocalization”. We note that a similar transient delocalization mechanism has been very recently proposed for exciton transport in polymeric nanofibers.^38,39^

### 3.3 Impact of Structural Disorder: Crystallinity–Mobility Relation

**Figure 8:**
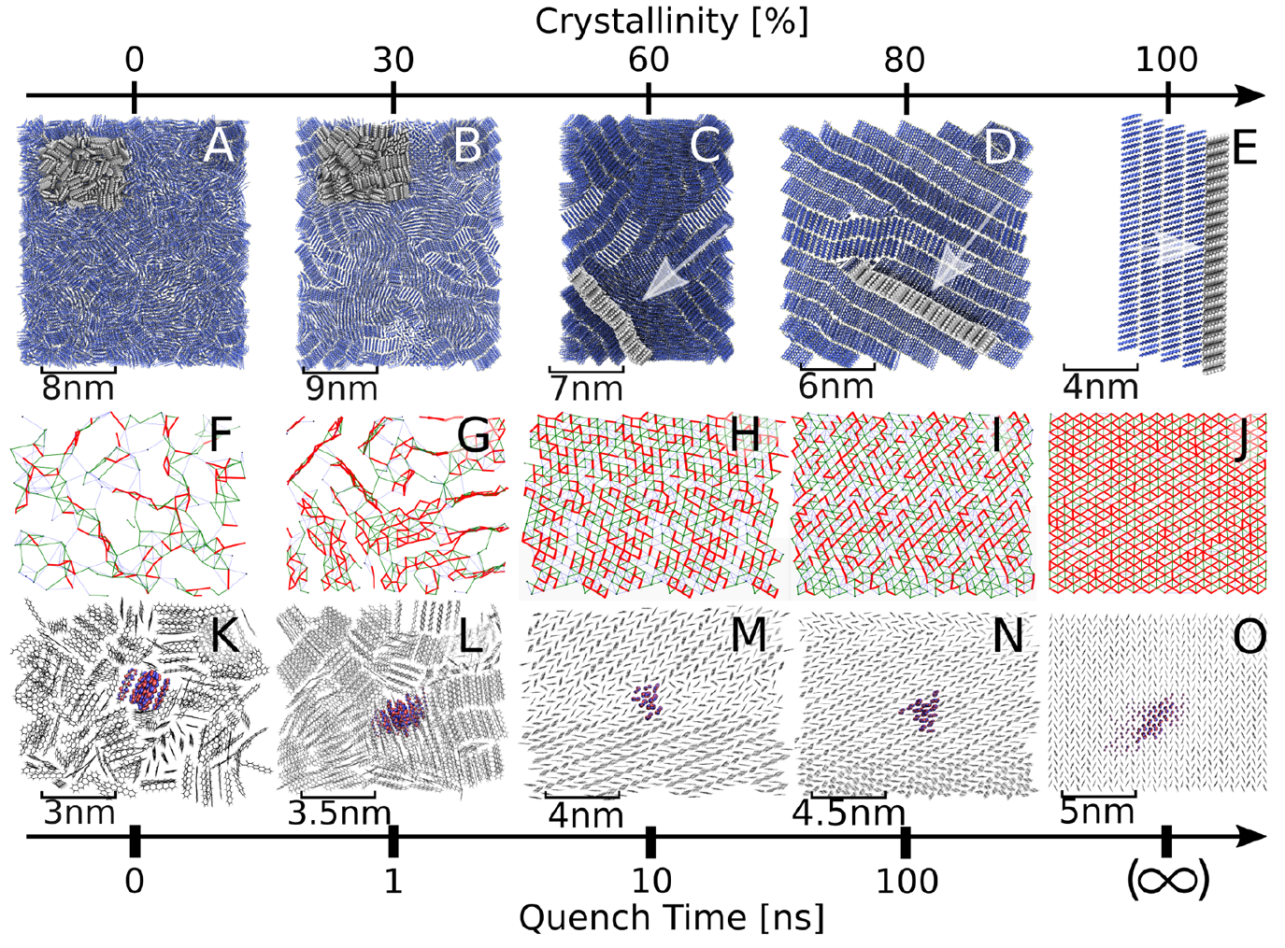
Structure (A–E), electronic coupling maps (F–J), and hole carrier wave function (K–O) of amorphous, nanocrystalline, and single crystalline pentacene phases. The crystallinity of the sample is obtained by linearly interpolating the mass density of the sample between the densities of amorphous (0%) and single crystalline phases (100%). See section 3.3 for further details. Reproduced from ref (4) with permission from Wiley.

We now turn to the question how charge transport is impacted by the different structural phases that one and the same molecule can form. To this end, we used a melt-quench procedure and varied the quenching time to generate a number of pentacene samples of increasing crystallinity, from fully amorphous to nanocrystalline (Figure 8, panels A–D). At the longer quenching times (10 and 100 ns), the pentacene molecules spontaneously self-assemble to nanocrystalline domains, each forming the characteristic herringbone layers of the single crystal, and separated by grain boundaries, as best visible in panels M and N.

**Figure 9:**
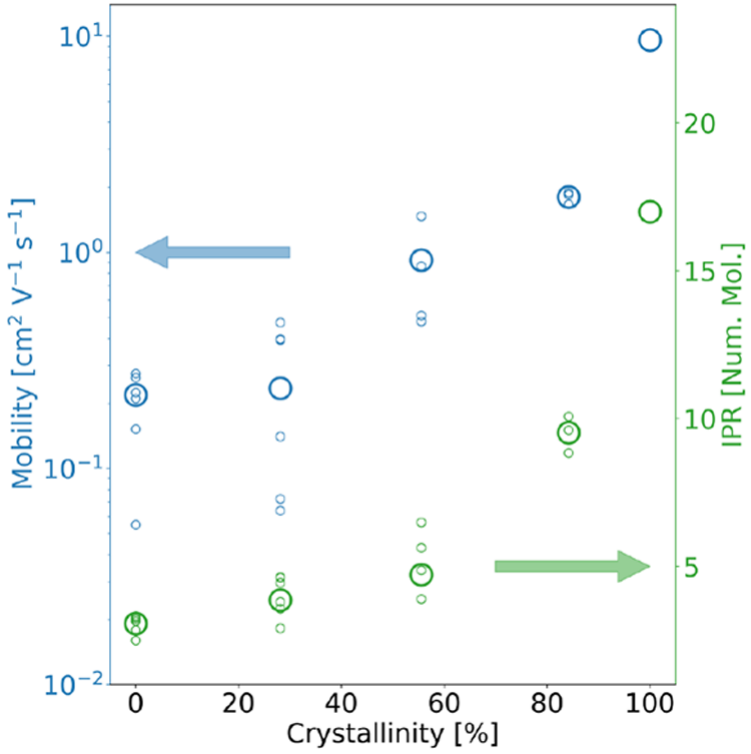
Hole mobilities and IPR for the pentacene phases shown in Figure 8, as obtained from FOB-SH simulation. The local mobilities and IPR for different regions of the samples are indicated in small circles and the averages are shown in large circles. Reproduced from ref (4) with permission from Wiley.

A first important observation is that the magnitude of the electronic coupling between different molecules is strongly impacted by the morphology of the system. As shown in Figure 8F–J, the number of strong electronic connections between the molecules (*V* > λ/2, i.e., ξ > 1, indicated in red) markedly increases as the crystallinity of the sample increases. FOB-SH simulation of charge carrier transport gave a good correlation between the crystallinity of the sample, the average size of the polaron (Figure 8K–O), and the mobility (Figure 9). In the amorphous sample, the transport occurs via a sequence of hops, whereas in the samples of higher crystallinity the mechanism resembles more closely the transient delocalization scenario in the single crystal (see section 3.2). Interestingly, the mobility at 80% crystallinity is only a fraction of the one for the single crystal: the transport is found to be limited by escape from grain boundaries, which act as carrier traps. For a more detailed discussion, we refer the interested reader to ref (4).

## 4 Better than Today’s Best OSs: Design Rules

As discussed in the Introduction, OSs with charge mobilities exceeding the values of today’s best materials would help improve the performance of existing devices and might lead to entirely new applications. In the following we review the design rules that could help achieve that.

Rule 0: *The material should be free of any localized and energetically low-lying electronic trap states introduced by undesired impurities or defects.* This is of course trivial, hence we call this Rule 0. If trap states are present, the charge carrier will occupy this state and the escape rate from it will be very slow (see Figure 9).^4^ Once this is achieved, one can start to increase the intrinsic mobility of the material. The results of both FOB-SH^1,3^ and TLT^7,22^ suggest, that any such attempt should focus on increasing the delocalization of the thermally accessible electronic states near the top of the valence band (hole transport) or the bottom of the conduction band (electron transport). The following rules aim to achieve that.

Rule 1: *The electronic coupling between nearest neighbors should exceed half of the reorganization energy, H*_*kl*_ > *λ/2.* If this is the case, the energy barriers for incoherent charge hopping no longer exist and charges delocalize over sites (molecules),^11,63^ see Figures 4−6.

Rule 2: *The strength of electronic coupling between nearest neighbors should be isotropic.* Both FOB-SH^3^ and TLT^7,22^ have shown that transport in 2D (or 3D) materials is more effective than transport in 1D materials. For typical molecular OSs forming conductive 2D planes, the charge mobility in a given direction within the plane was found to increase by a factor of 2–3 when the simulations were carried out for the full 2D planes rather than for 1D models, see Table 1 (“μ (2D)” versus “μ (1D)”). However, the extent of this effect depends on the sign combination of electronic couplings in the different directions (see below).

Rule 3: *The thermal fluctuations of electronic coupling and site energies (i.e., local and nonlocal electron–phonon coupling) should be minimized.* The width of the fluctuations should ideally be an order of magnitude smaller than the mean values; in many OSs the width is 1/3–1/2 of the mean value or even larger.^1,3,64,65^ These fluctuations cause disorder in the electronic Hamiltonian which gives rise to localized eigenstates at the band edges.^3^

Rule 4: *The electronic couplings between nearest neighbors should have a favorable sign combination.* Assuming that the OSs can be described by three distinct nearest neighbor couplings, the electronic states at the band edges tend to be delocalized when the product of the couplings is positive (negative) for holes (electrons), and localized otherwise.^3,7,22^ This rule is a direct consequence of the symmetry of the system (and its Hamiltonian) which can be mapped onto a sphere as done in ref (22). According to this map, delocalization of the thermally accessible states is maximized when the couplings are all the same in different directions and present a positive (negative) sign combination for hole (electron). However, there are many OSs where more than three coupling values are reasonably large and need to be considered (e.g., perylene^3^); rules for these cases have not been established yet, to our knowledge.

Several experimental approaches have been pursued in the last two decades to increase charge mobilities along these lines, with reasonable success. Examples include decreasing defect concentration/energetic disorder by purification^66^ (Rule 0), application of gentle external pressure^67^ (Rules 1 and 3), minimizing intrachain disorder of polymers,^68^ and increasing resilience to thermal disorder by insertion of bulky side chains^69^ (Rule 3). In practice, a potential problem of property engineering is that improving one feature (e.g., decreasing thermal fluctuations of electronic couplings) could lead to the deterioration of another (e.g., decrease in mean electronic couplings).

Alternatively, in silico screening of crystallographic databases could help identify OSs that satisfy most of the design rules simultaneously. Rules 1, 2, and 4 can be investigated with standard DFT calculations on molecular dimers extracted from the material.^70−73^ Rule 1 has been used in our group to identify high-mobility tetracene derivatives via DFT calculations, followed by FOB-SH mobility prediction.^42^ Remarkably, we found that only a small change in the length of the tetracene side chains, specifically a modification of an ethyl to a methyl group, results in an increase in the hole mobility along the columnar stacks from 0.6 to 20.8 cm^2^ V^–1^ s^–1^ (as a consequence of a small change in the packing structure that led to an increase in orbital overlap and electronic coupling).^42^ Rule 3 requires either the calculation of the full off-diagonal electron–phonon coupling constants or MD simulation. The latter is impractical for screening studies, and the former is extremely expensive at the DFT level but feasible with lower-level or approximate methods.^71,72^

**Figure 10:**
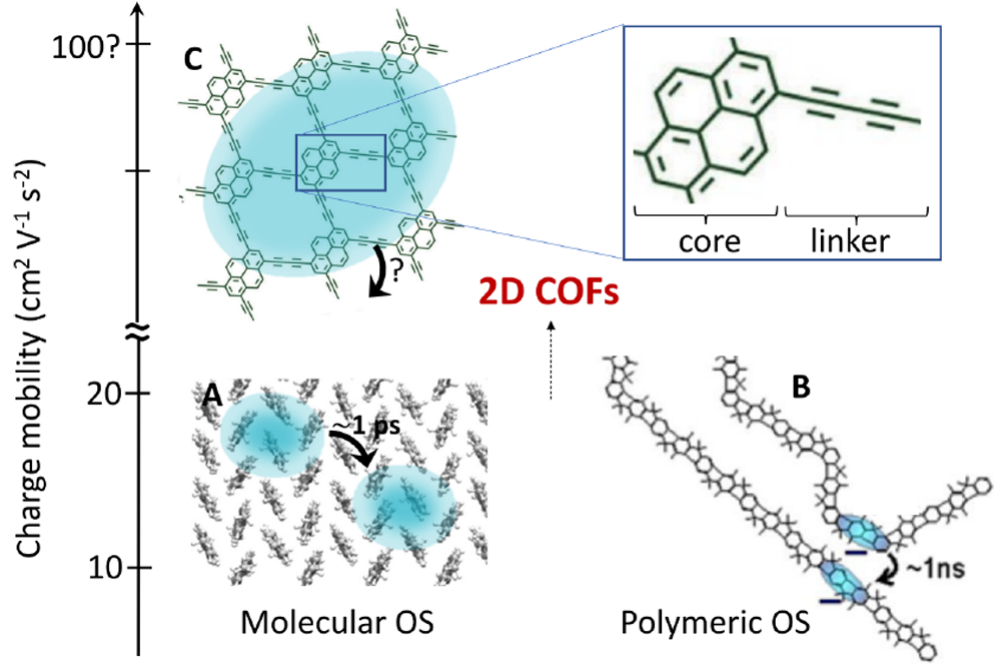
Promise of 2D covalent organic framework materials (COFs). Panel (b) modified from ref (7) with permission from Nature Publishing Group. Panel (c) modified from ref (77) with permission from The Royal Society of Chemistry.

Two-dimensional covalent organic frameworks (2D COFs) appear as a promising new class of electronic materials that could push charge mobility of OSs to new heights (see Figure 10). This is because 2D COFs combine several important features that are beneficial toward fulfilling the above-mentioned design rules: the covalent linking of organic molecules gives rise to “through-bond” electronic couplings between the cores that are likely to be higher than the through-space couplings in molecular crystals (Rule 1), while, at the same time, their thermal fluctuations are expected to be smaller due to the rigidity of the cross-linked network (Rule 3). Moreover, the couplings within the planes are isotropic by design (Rule 2) and the sign combination is likely to be favorable as well (Rule 4). Although a number of computational studies have been carried out on 2D COFs,^74−76^ the physics of charge transport in these intriguing materials is still relatively poorly understood, both theoretically and in terms of experimental characterization.

## 5 Conclusions and Prospects

In this Account, a novel nonadiabatic molecular dynamics approach, termed FOB-SH, for the direct time propagation of charge carriers in “soft” materials was reviewed. The electronic structure of the material is coarse-grained by employing an extended tight-binding electronic Hamiltonian with matrix elements parametrized to explicit electronic structure calculations and updated on-the-fly along the trajectories. This makes FOB-SH computationally extremely efficient permitting the simulation of charge transport in truly nanoscale systems of more than 1000 organic molecules (on the order of 1 ps/day/trajectory on a single compute core).

FOB-SH gives unprecedented insight into the transport mechanism of charge carriers in organic materials, and it provides a molecular interpretation of the dichotomic nature of charge carriers as inferred from experimental measurements as well as numerical support for the assumptions of the recently proposed transient localization theory. Our simulation approach covers a wide range of transport regimes, from small polaron hopping to large polaron diffusion, predicts rather than assumes the charge transport mechanism, yields charge mobilities in good agreement with available experimental data, and can be applied to ordered and disordered samples. However, as with any quantum-classical method, certain nuclear quantum effects are not included; hence, caution is warranted for applications at low temperatures. In addition, applications to new/more complex organic materials will require the development of reliable force fields.

Future applications of FOB-SH might give a better understanding of charge transport in the fascinating class of 2D COF materials. Moreover, the generality of our approach lends itself to the simulation of other transport and electronic relaxation phenomena of interest in OSs, for instance, Frenkel exciton delocalization and transport, exciton dissociation and recombination. Such simulations could guide the development of next-generation soft optoelectronic materials with minimal energetic penalty, which is expected to benefit organic photovoltaics as well as other applications where as much photon energy as possible needs to be retained in the charge separated states, including organic photocatalysis and artificial photosynthesis.
